# Experiences with and impacts of the COVID-19 pandemic by substance use disorder in the early phase of pandemic in the United States: A cross-sectional survey, 2020

**DOI:** 10.1371/journal.pone.0271788

**Published:** 2022-07-21

**Authors:** Andrea Acevedo, Wenhui Feng, Laura Corlin, Jennifer D. Allen, Peter Levine, Thomas J. Stopka

**Affiliations:** 1 Department of Community Health, Tufts University, Medford, Massachusetts, United States of America; 2 Department of Public Health and Community Medicine, Tufts University School of Medicine, Boston, Massachusetts, United States of America; 3 Department of Civil and Environmental Engineering, Medford, Massachusetts, United States of America; 4 Tisch College of Civic Life, Tufts University, Medford, Massachusetts, United States of America; 5 Clinical and Translational Sciences Institute, Tufts University School of Medicine, Boston, Massachusetts, United States of America; Wachemo University, ETHIOPIA

## Abstract

**Introduction:**

The coronavirus disease 2019 (COVID-19) pandemic could disproportionately affect individuals who have a substance use disorder (SUD). However, little information exists on COVID-19-related experiences among individuals with a SUD. We examined whether individuals with a SUD differ from other individuals with regard to COVID-19 testing, susceptibility, and employment-related vulnerability.

**Methods:**

We used data from a U.S. nationally representative survey (n = 1,208). Using logistic regressions, we examined whether individuals with SUDs differ from other individuals regarding underlying health conditions, COVID-19 testing, access to paid sick leave, and loss of employment. Data were collected in late May-early June, 2020.

**Results:**

Four percent of participants reported that a healthcare professional had told them they had a SUD. We found that, compared to those without SUDs, respondents with SUDs had higher odds of having lost their job due to the pandemic (adjusted odds ratio [AOR]:5.17, 95% confidence interval [CI]:2.28–11.74). Among individuals who were employed prior to the pandemic, people with SUDs had lower odds of having paid sick leave (AOR:0.26, 95% CI:0.09–0.74).

**Conclusion:**

Our study indicates that individuals with SUDs could be disproportionately affected by COVID-19 economically, which might worsen SUD and racial/ethnic health disparities.

## Introduction

There is growing concern about the impact of the coronavirus (COVID-19) pandemic on the health of individuals who have a substance use disorder (SUD) [1]. Individuals with a SUD might also be disproportionately affected by the rise in pandemic-related unemployment [2] since some industries that were highly impacted by the shutdowns, such as food service, are more common among those who report illicit substance use [3].

Despite these concerns, very little data exist on COVID-19 testing and pandemic-related challenges among individuals with a SUD [4, 5]. In the current study, we surveyed a nationally representative sample of adults in the United States (U.S.) in the early stages of the pandemic to explore differences between individuals with and without a SUD with regard to: underlying health conditions that exacerbate COVID-19 morbidity, COVID-19 testing, available paid sick leave, and loss of employment.

## Methods

### Data source and study-specific post-stratification weights

We analyzed data from the Equity in Health, Wealth, and Civic Engagement survey conducted by Tufts University [6]. The survey was fielded by Ipsos using the web-enabled KnowledgePanel, the largest online probability-based panel designed to be representative of the U.S. population. KnowledgePanel members were recruited using address-based probability sampling, and for those who agreed to participate, Ipsos provided a laptop/netbook and internet access at no cost to the participants if they do not have it.

KnowledgePanel members represented the adult population in the U.S. with respect to a broad set of geodemographic indicators, as well as hard-to-reach adults (such as those without internet access or Spanish-language-dominant Hispanics) who are recruited in representative proportions. Thus, the raw distribution of KnowledgePanel mirrors that of the U.S. adults relatively closely, barring occasional disparities that may emerge for certain subgroups due to differential attrition.

To select the general population sample for the present study, Ipsos used its patented methodology, which initially focuses on weighting the pool of active members to the geodemographic benchmarks secured from the March 2019 supplement of the U.S. Census Bureau’s Current Population Survey along the geodemographic dimensions listed below [7]. Using the resulting weights as measures of size, a probability-proportional-to-size procedure was used to select the study sample. The geodemographic dimensions used to weight the active panel members for computation of size measures included: gender; age; race/ethnicity; educational attainment; census region; annual household income; home ownership status; a metropolitan area indicator; and Hispanic origin.

Once all survey data were collected and processed, design weights were adjusted to account for differential nonresponse. Using geodemographic distributions obtained from the Community Population Survey and the U.S. Census Bureau’s American Community Survey (ACS), we applied an iterative proportional fitting (raking) procedure to produce the final weights. We used the following benchmark distributions of U.S. adults aged 18 and older from the most recent Community Population Survey March Supplement (2019) for the ranking adjustment of weights, and we used the 2018 ACS language proficiency benchmarks to adjust weights for Hispanic respondents: gender by age; race/ethnicity; census region by metropolitan status; education; annual household income; and language proficiency. In the last step, we examined calculated weights to identify outliers at the extreme lower and upper tails of the weight distribution, and we determined that no pruning of outliers was needed. The resulting weights were then scaled to aggregate to the total sample size of all eligible respondents. The design effect was 1.2487.

The survey was fielded with panel members in English and Spanish from May 29^th^ to June 10^th^, 2020. Upon completion, qualified respondents received their standard incentive payment (for most, 1000 points, the cash-equivalent of $1), and an entry into a raffle. The study was approved by the Social and Behavioral Research Institutional Review Board at Tufts University (Approval #: STUDY 00000428). Written consent to participate in the Equity in Health, Wealth, and Civic Engagement survey was obtained online.

### Sample

A random sample of 1,980 panel members was selected for recruitment for the survey and 1,267 participants responded to the invitation, yielding a final stage completion rate of 64.0%. We excluded anyone who had missing data on our main variable of interest, SUD (n = 59), yielding a final analytical sample of n = 1,208.

### Variables

Demographic measures and questions related to existing health conditions were drawn from the Ipsos Core Profile and Health Surveys. Questions related to COVID-19 and substance use disorders were part of the “Equity in Health, Wealth, and Civic Engagement” survey. These questions were developed specifically for the survey. The survey was pre-tested in both languages, with a total sample of 83 respondents drawn from the Ipsos universe. We provide the English and Spanish versions of the questions in the S3 Appendix.

#### Main independent variable

Participants were asked whether a health care professional had told them they had a SUD, an opioid use disorder, or an alcohol use disorder. An affirmation to any of these questions was coded as a SUD.

#### Dependent variables

We focused on four dependent variables: 1) We examined whether the participant had several of the underlying conditions identified by the Centers for Disease Control and Prevention as conditions that might place individuals at increased risk for severe illness from COVID-19 regardless of age [8] (See conditions included in S1 Appendix). The questions were modified from questions from the National Health Interview Survey. Our dependent variable was a dichotomous variable indicating whether participants reported any of these conditions; 2) Participants were asked whether they had been tested for COVID-19; 3) Participants who had been employed prior to the pandemic were asked whether their employer provided paid sick leave; 4) Among participants who were not retired, they were asked whether they had been laid off due to the pandemic. Missing data for our dependent variables ranged from 0–3% and did not vary significantly by SUD status.

#### Covariates

Our covariates were selected *a priori* based on their association with COVID-19 and SUDs: gender [men/women], age [continuous], geographic region [Northeast, South, Southwest, West], and annual household income [<$40,000; $40,000-$99,999; $100,000+]. We also included insurance status [Employer-based, Government (Medicare/Medicaid/Veterans Health Administration) or marketplace, Other, or None] based on recent findings that the relationship between having an opioid use disorder and COVID-19 related outcomes varied by insurance status [4]. These items were modified versions of questions used in the American Community Survey or America National Election Studies.

### Analyses

We conducted chi-squared tests and a Wald test adjusting for survey structure to assess bivariate associations between demographic characteristics and SUD status. We constructed separate bivariate and multivariable logistic regression models for the association between SUD and each dependent variable. We also conducted additional analyses using the same outcomes. We conducted the logistic regression models stratifying the sample by gender. We conducted the logistic regression models with alcohol use disorder (AUD) status as the main independent variable. Similarly, we conducted the logistic regression models with opioid use disorder (OUD) as the main independent variable. Survey weights were applied in the analyses.

## Results

Approximately 4% of the sample reported having a SUD. There were no significant differences in demographic characteristics by SUD status at the α = 0.05 level, except that those with SUD were significantly younger (Mean = 43.3, SD = 16.2) than those without a SUD (Mean = 48.9, SD = 17.5) (Table 1). Two-thirds (68%) of survey respondents reported underlying health conditions that place them at higher risk for COVID-19 complications. COVID-19 testing was reported by 6.4% of respondents. Nearly seven in ten respondents (67%) who were employed reported having a job that provided paid sick leave and about 17% reported being laid off due to the pandemic.

**Table 1:** Survey participant characteristics by SUD status (%^a^, unless otherwise specified), U.S., May 29-June 10 2020, (n = 1,208).

Variable	Overall (n = 1,208)	No SUD (n = 1,163)	SUD (n = 45)	p-value^b^
**Age (mean, standard deviation)**	48.7 (17.5)	48.9 (17.5)	43.3 (16.2)	0.04
**Gender**				
Men	47.2	46.7	58.8	0.17
Women	52.8	53.3	41.2	
**Region**				
Midwest	20.6	20.8	17.0	0.79
Northeast	17.5	17.3	22.8	
South	38.2	38.7	34.2	
West	23.7	23.2	26.0	
**Annual Income**				
<$40K	24.3	28.6	24.5	0.85
$40K-$99,999	38.2	33.1	38.1	
$100,000+	37.4	35.4	37.4	
Health insurance				
Employer based	50.5	51.1	35.4	0.06
Government or Marketplace	31.9	31.2	49.9	
Other^c^	2.8	2.9	0.0	
None	6.6	6.4	12.2	
Missing/refused	8.2	8.5	2.6	
**Race/ethnicity**				
Non-Hispanic White	63.7	63.2	74.2	0.20
Non-Hispanic Black	11.7	12.2	0.0	
Hispanic	16.4	16.4	12.2	
Another race/ethnicity	6.8	6.9	5.88	
Multiracial	1.4	1.3	2.8	

SUD = Substance Use Disorders

**Note**:

^a^Percentages reflect survey weighting to be representative of the non-institutionalized U.S. population.

^b^Differences tested using Chi-squared tests or a Wald test.

^c^Given the low proportion of Other sources of health insurance and that none of the participants with other health insurance had an SUD, we combined this category with missing/refused in the regression models.

Associations between SUD and each outcome measure estimated in separate models did not change substantially after adjusting for key demographic characteristics (Table 2). We observed that participants with SUD were 74% less likely to have paid sick leave (adjusted odds ratio [AOR]: 0.26, 95% confidence interval [CI]: 0.09–0.74) and were about five times as likely to have been laid off due to the COVID-19 pandemic (AOR: 5.17; 95% CI: 2.28–11.74). Whereas respondents with SUD showed trends towards increased odds of having underlying conditions and increased odds for being tested for COVID-19, associations were not significant for either of these outcomes.

**Table 2:** Crude and adjusted associations between substance use disorders and COVID-related outcomes, May 29-June 10, 2020.

	Outcomes			
	Have 1+ underlying conditions that could make COVID-19 severe^a^ (N = 1,189; n = 853)^b^	Had COVID-19 test (N = 1,185; n = 76) ^b^	Have paid sick leave^c^ (N = 659; n = 440) ^b^	Laid off because of COVID-19 pandemic^d^ (N = 964; n = 137) ^b^
	OR (95% CI)	OR (95% CI)	OR (95% CI)	OR (95% CI)
**SUD (Yes vs. No) (unadjusted)**	1.65 (0.72–3.78)	2.00 (0.71–5.60)	**0.23 (0.09–0.60)**	**6.07 (2.80–13.15)**
**SUD (Yes vs. No) (adjusted)** ^e^	1.97 (0.81–4.78)	2.15 (0.72–6.46)	**0.26 (0.09–0.74)**	**5.17 (2.28–11.74)**

SUD = Substance use disorder

Note:

^a^Participant is obese or a current or former smoker, or reported that a clinician had diagnosed them with one or more of the following underlying health conditions: hypertension, had a respiratory disease, diabetes, kidney disease, heart disease, pulmonary hypertension, liver disease, or HIV/AIDS.

^b^N = weighted total sample size with data in outcomes; n = total number of respondents who experienced the outcome;

^c^Among employed individuals;

^d^Excluding retired individuals;

^e^Adjusting for gender (men/women), age (continuous), geographic region (Northeast, South, Southwest West), annual household income (<$40,000; $40,000-$99,999; $100,000+), insurance (employer-based, government/marketplace, other/missing, none).

**Bold** = statistically significant at p < 0.05 or lower

When we stratified the sample by gender, we found that results were similar for men and women with a few exceptions. Women with a SUD were estimated to be almost eight times more likely to have had a COVID-19 test (AOR: 7.73; 95% CI: 2.00–29.86) compared to women without a SUD; whereas, there was no association between SUD status and COVID-19 testing among men. Women with a SUD were no more likely than women without a SUD to have paid sick leave (AOD: 0.80, 95% CI: 0.14–4.76). However, among men, those with a SUD were significantly less likely to have paid sick leave compared to those without a SUD (See Tables A and B in S2 Appendix).

We also examined whether results varied by type of substance use disorder. The results of these analyses should be interpreted cautiously due to the small number of cases. Approximately 3% of the sample reported having been diagnosed with an alcohol use disorder (AUD). When we examined the association between AUD and our outcomes, we found that individuals with an AUD were 77% less likely to have paid sick leave (AOR = 0.23, 95% CI: 0.06–0.84), and almost six times more likely to have been laid off due to COVID-19 (AOR = 5.78, 95% CI: 2.15–15.54) relative to those without an AUD. Unlike our general findings, individuals with an AUD were significantly more likely to have had a COVID-19 test (AOR: 3.39, 95% CI: 1.11–10.41) (See Table C in S2 Appendix). There was no significant association between AUD and having underlying conditions that could make COVID-19 more severe. Only 1% of the sample reported having been diagnosed with an opioid use disorder (OUD). Participants with an OUD were estimated to be about nine times more likely to have been laid off during the pandemic (AOR = 9.25, 95% CI: 2.46–34.80). None of the associations between OUD and the other outcomes were statistically significant. (See Table D in S2 Appendix).

## Discussion

We conducted one of the first nationally representative studies focused on associations between SUD and COVID-19 experiences in the U.S. We found that individuals with a SUD had decreased odds of having access to paid sick leave and increased odds of being laid off from work due to the pandemic. Recent studies have found that individuals with an SUD were at increased risk for contracting COVID-19, and among those who contract COVID-19, those with SUDs have worse outcomes including higher risk of hospitalization and death [4, 5]. The present study highlights important non-clinical impacts of the pandemic affecting individuals with SUD and point to structural inequities that can amplify the effects of the pandemic in this population.

Our finding that having a SUD was associated with being less likely to have paid sick leave suggests that people with SUD were at higher risk of losing income if they were to get COVID-19. The Families First Coronavirus Response Act, passed in March 2020, required employers with <500 employees to provide up to 80 hours of paid sick time to employees who had COVID-19 symptoms and/or those who needed to quarantine due to COVID-19 [9]. These benefits expired at the end of 2020. As these benefits were not extended, COVID-19 could further exacerbate health and economic disparities among those with a SUD.

Our study also suggests that individuals with SUD might have been disproportionately affected by the rise in unemployment during the early months of the pandemic. This finding could be due to differences in the sectors within which individuals with SUDs are more likely to be employed, or occupational sectors where workers are at higher risk of SUDs. Among adults who report illicit substance use, the three most common industries they work in include industries particularly affected by the COVID-19 shutdown: accommodations; food services; and arts, entertainment, and recreation [3].

In addition to economic consequences, unemployment is associated with increased alcohol and drug use [10], which could be especially detrimental to individuals with SUDs and those in recovery. Additionally, loss of income and loss of employment-based health insurance will likely increase barriers to SUD treatment, at a time when the need for treatment is even greater [11, 12]. Furthermore, since Black and Latino individuals have been disproportionately impacted by COVID-19 [13] and by the increase in unemployment [2], and already have lower access to SUD treatment [14], there is reason to believe that these events will exacerbate racial/ethnic health inequities.

Our findings have several implications. At a time when overdose deaths are at an all-time high [15], it is critical that the needs of people with SUD are addressed. Health care providers working with patients with a SUD should consider assessing how COVID-19 has impacted their patients’ employment situation. If patients have been negatively affected, providers should connect their patients to community and state resources and wraparound services that provide financial and non-financial supports for the unemployed in general, and specialized services for patients with SUDs (e.g., referrals to social safety-net programs; employment and training programs; SUD treatment and support systems; and provision of a prescription for medication for opioid use disorder, in particular). Given our finding that individuals with SUD who are employed are less likely to have paid sick leave, SUD treatment facilities could provide COVID-19 vaccinations on-site to facilitate immunization among people with SUD.

Ultimately, policy change is needed to help address the impact that the pandemic is having on individuals with SUDs. Policymakers, for example, could require employers to provide paid sick time. Such a policy would support individuals regardless of SUD status, but could be especially beneficial to individuals with SUD. Policymakers should also work to improve accessibility of SUD treatment services given the documented connection between unemployment and substance use. During the COVID-19 pandemic, a number of restrictions have been lifted for SUD treatment providers, including removing the requirement of an in-person evaluation for patients to begin buprenorphine treatment, options for telemedicine-based treatment, and longer take homes for methadone [16]. Continuation of these relaxed policies, even when/if the pandemic subsides, are worthy of consideration.

Although research is growing on the impact of the pandemic on individuals with a SUD, more research is needed on the understanding the long-term health and social impacts of the pandemic on individuals with SUD, and on interventions at the provider, community, and national levels to ameliorate the negative consequences of the pandemic on an already vulnerable population.

A limitation of the study is that we used a measure of SUD based on having been diagnosed with a SUD. To the extent that SUDs are underdiagnosed by health care professionals [17], our SUD variable is likely an underestimate of the true prevalence of SUDs. Future studies should use additional sources of data (e.g., medical records, health services). We did not have large enough sample size in most racial/ethnic groups to examine the differential impact of SUDs on our outcomes by race/ethnicity, particularly given the low prevalence of SUD. Our sample included only 4% of individuals who met our measure of SUD. Larger studies with more participants who have a SUD and that include a sample that is more racially/ethnically diverse are needed to understand physical, mental, economic, and social consequences of COVID-19 among individuals with a SUD.

## Conclusions

Individuals with SUDs are disproportionately affected by COVID-19 in employment. Policy makers should work to further expand access to SUD treatment and address the economic impact the pandemic is having.

## Supporting information

S1 Appendix Health conditions that could exacerbate COVID-19 morbidity. (DOCX)

S2 Appendix Additional analyses. (DOCX)

S3 Appendix Item questions used in study. (DOCX)
