# Supplementary material for: Experiences with and impacts of the COVID-19 pandemic by substance use disorder in the early phase of pandemic in the United States: A cross-sectional survey, 2020
Source: PLoS One. 2022 Jul 21;17(7):e0271788. doi: 10.1371/journal.pone.0271788 (PMC9302744; doi:10.1371/journal.pone.0271788)
Supplement: S1 Appendix — (DOCX) [file pone.0271788.s001.docx]

**S1 Appendix. Health conditions that could exacerbate COVID-19 morbidity.**

We examined whether the participant was obese (i.e., body mass index (BMI) of ≥ 30); self-reported smoking; and that a clinician diagnosed underlying health conditions (one or more of: hypertension, a respiratory disease [e.g., chronic obstructive pulmonary disease, asthma], diabetes, kidney disease, heart disease, pulmonary hypertension, liver disease [hepatitis C virus or non-alcoholic fatty liver disease], or HIV/AIDS). These underlying conditions are identified by the Centers for Disease Control and Prevention (CDC) as conditions that might place individuals at increased risk for severe illness from COVID-19, regardless of age.^15^ We used the guidelines updated in July, 2020.
