# Supplementary material for: Experiences with and impacts of the COVID-19 pandemic by substance use disorder in the early phase of pandemic in the United States: A cross-sectional survey, 2020
Source: PLoS One. 2022 Jul 21;17(7):e0271788. doi: 10.1371/journal.pone.0271788 (PMC9302744; doi:10.1371/journal.pone.0271788)
Supplement: S2 Appendix — (DOCX) [file pone.0271788.s002.docx]

**S2 Appendix. Additional Analyses.**

**Table A. Crude and adjusted associations between substance use disorders and COVID-related outcomes, May 29-June 10, 2020 – Males only**

|  | **Outcomes** | | | |
| --- | --- | --- | --- | --- |
|  | **Have 1+ underlying conditions that could make COVID-19 severe^a^**  **(N =561; n = 426)^b^** | **Had COVID-19 test**  **(N =560; n = 41) ^b^** | **Have paid sick leave^c^**  **(N = 335; n = 227) ^b^** | **Laid off because of COVID-19 pandemic^d^**  **(N = 451; n = 69) ^b^** |
|  | **OR (95% CI)** | **OR (95% CI)** | **OR (95% CI)** | **OR (95% CI)** |
| **SUD (Yes vs. No) (unadjusted)** | 1.98  (0.64 -6.17) | 0.31  (0.04 – 2.40) | **0.15**  **(0.05-0.52)** | **5.42**  **(2.03-14.48)** |
| **SUD (Yes vs. No)**  **(adjusted)^e^** | 2.53  (0.80-8.01) | 0.32  (0.04-2.72) | **0.16**  **(0.05-0.51)** | **4.52**  **(1.44-14.14)** |

**SUD= Substance use disorder**

**Note:** ^a^Participant is obese or a current or former smoker, or reported that a clinician had diagnosed them with one or more of the following underlying health conditions: hypertension, had a respiratory disease, diabetes, kidney disease, heart disease, pulmonary hypertension, liver disease, or HIV/AIDS. ^b^N = weighted total sample size with data in outcomes; n = total number of respondents who experienced the outcome; ^c^Among employed individuals; ^d^Excluding retired individuals; ^e^Adjusting for gender (men/women), age (continuous), geographic region (Northeast, South, Southwest West), annual household income (<$40,000; $40,000-$99,999; $100,000+), insurance (employer-based, government/marketplace, other/missing, none). **Bold** = statistically significant at p < 0.05 or lower

**Table B. Crude and adjusted associations between substance use disorders and COVID-related outcomes, May 29-June 10, 2020 – Females only**

|  | **Outcomes** | | | |
| --- | --- | --- | --- | --- |
|  | **Have 1+ underlying conditions that could make COVID-19 severe^a^**  **(N =628; n = 427)^b^** | **Had COVID-19 test**  **(N =626; n = 35) ^b^** | **Have paid sick leave^c^**  **(N = 324; n = 213) ^b^** | **Laid off because of COVID-19 pandemic^d^**  **(N = 503; n =68) ^b^** |
|  | **OR (95% CI)** | **OR (95% CI)** | **OR (95% CI)** | **OR (95% CI)** |
| **SUD (Yes vs. No) (unadjusted)** | 1.30  (0.38 - 4.47) | **5.93**  **(1.68 – 20.93)** | 0.58  (0.11-3.16) | **7.25**  **(2.08-25.36)** |
| **SUD (Yes vs. No)**  **(adjusted)^e^** | 1.60  (0.39-6.52) | **7.73**  **(2.00-29.86)** | 0.80  (0.14-4.76) | **6.28**  **(1.77-22.29)** |

**SUD= Substance use disorder**

**Note:** ^a^Participant is obese or a current or former smoker, or reported that a clinician had diagnosed them with one or more of the following underlying health conditions: hypertension, had a respiratory disease, diabetes, kidney disease, heart disease, pulmonary hypertension, liver disease, or HIV/AIDS. ^b^N = weighted total sample size with data in outcomes; n = total number of respondents who experienced the outcome; ^c^Among employed individuals; ^d^Excluding retired individuals; ^e^Adjusting for gender (men/women), age (continuous), geographic region (Northeast, South, Southwest West), annual household income (<$40,000; $40,000-$99,999; $100,000+), insurance (employer-based, government/marketplace, other/missing, none). **Bold** = statistically significant at p < 0.05 or lower

**Table C. Crude and adjusted associations between alcohol use disorders and COVID-related outcomes, May 29-June 10, 2020**

|  | **Outcomes** | | | |
| --- | --- | --- | --- | --- |
|  | **Have 1+ underlying conditions that could make COVID-19 severe^a^**  **(N =1,188; n = 864)^b^** | **Had COVID-19 test**  **(N =1,184; n = 78) ^b^** | **Have paid sick leave^c^**  **(N = 657; n = 445) ^b^** | **Laid off because of COVID-19 pandemic^d^**  **(N = 963; n = 136) ^b^** |
|  | **OR (95% CI)** | **OR (95% CI)** | **OR (95% CI)** | **OR (95% CI)** |
| **AUD (Yes vs. No) (unadjusted)** | 2.45  (0.89 -6.74) | **3.04**  **(1.06-8.74)** | **0.18**  **(0.06-0.55)** | **6.42**  **(2.58-16.00)** |
| **AUD (Yes vs. No)**  **(adjusted)^e^** | 2.84  (0.91-8.84) | **3.39**  **(1.11-10.41)** | **0.23**  **(0.06-0.84)** | **5.78**  **(2.15-15.54)** |

**AUD= Alcohol use disorder**

**Note:** ^a^Participant is obese or a current or former smoker, or reported that a clinician had diagnosed them with one or more of the following underlying health conditions: hypertension, had a respiratory disease, diabetes, kidney disease, heart disease, pulmonary hypertension, liver disease, or HIV/AIDS. ^b^N = weighted total sample size with data in outcomes; n = total number of respondents who experienced the outcome; ^c^Among employed individuals; ^d^Excluding retired individuals; ^e^Adjusting for gender (men/women), age (continuous), geographic region (Northeast, South, Southwest West), annual household income (<$40,000; $40,000-$99,999; $100,000+), insurance (employer-based, government/marketplace, other/missing, none). **Bold** = statistically significant at p < 0.05 or lower

**Table D. Crude and adjusted associations between opioid use disorders and COVID-related outcomes, May 29-June 10, 2020**

|  | **Outcomes** | | | |
| --- | --- | --- | --- | --- |
|  | **Have 1+ underlying conditions that could make COVID-19 severe^a^**  **(N =1,189; n = 876)^b^** | **Had COVID-19 test**  **(N =1,185; n = 77) ^b^** | **Have paid sick leave^c^**  **(N = 659; n = 448) ^b^** | **Laid off because of COVID-19 pandemic^d^**  **(N = 964; n = 137) ^b^** |
|  | **OR (95% CI)** | **OR (95% CI)** | **OR (95% CI)** | **OR (95% CI)** |
| **OUD (Yes vs. No) (unadjusted)** | 2.32  (0.36 -14.93) | 1.85  (0.22 – 14.55) | 0.44  (0.07-2.71) | **12.51**  **(3.15-49.63)** |
| **OUD (Yes vs. No)**  **(adjusted)^e^** | 3.82  (0.52-28.00) | 1.93  (0.21-18.13) | 0.70  (0.86-4.39) | **9.25**  **(2.46-34.80)** |

**OUD= Opioid use disorder**

**Notes:** ^a^Participant is obese or a current or former smoker, or reported that a clinician had diagnosed them with one or more of the following underlying health conditions: hypertension, had a respiratory disease, diabetes, kidney disease, heart disease, pulmonary hypertension, liver disease, or HIV/AIDS. ^b^N = weighted total sample size with data in outcomes; n = total number of respondents who experienced the outcome; ^c^Among employed individuals; ^d^Excluding retired individuals; ^e^Adjusting for gender (men/women), age (continuous), geographic region (Northeast, South, Southwest West), annual household income (<$40,000; $40,000-$99,999; $100,000+), insurance (employer-based, government/marketplace, other/missing, none). **Bold** = statistically significant at p < 0.05 or lower
