# Supplementary material for: Experiences with and impacts of the COVID-19 pandemic by substance use disorder in the early phase of pandemic in the United States: A cross-sectional survey, 2020
Source: PLoS One. 2022 Jul 21;17(7):e0271788. doi: 10.1371/journal.pone.0271788 (PMC9302744; doi:10.1371/journal.pone.0271788)
Supplement: S3 Appendix — (DOCX) [file pone.0271788.s003.docx]

**S3 Appendix. Item Questions Used in Study**

**Questions that are part of IPSOS Knowledge Panel (English)**

| **Concept** | **Question** |
| --- | --- |
| **Gender** | Are you…?  1. Male  2. Female |
| **Employment Status** | Do any of the following currently describe you?  1. Employed full time (35 hours or more per week) for pay with an organization or company  2. Employed part time (less than 35 hours per week) for pay with an organization or company  3. Self-employed full time (35 hours or more per week)  4. Self-employed part time (less than 35 hours per week)  1. Yes  2. No  Do any of the following currently describe you?  1. Looking for work  2. Unable to work due to a disability or work-related injury  3. On temporary layoff from a job  1. Yes  2. No  Do any of the following currently describe you?  1. Retired  2. A student  3. A stay-at-home spouse or partner  4. Working in an unpaid job, such as an internship or volunteer position  5. Working as a freelancer or independent contractor for pay  1. Yes  2. No |
| **Household Income** | How much is the combined income of all members of YOUR HOUSEHOLD for the PAST 12 MONTHS?  Please include your income PLUS the income of all members living in your household (including cohabiting partners and armed forces members living at home). Please count income BEFORE TAXES and from all sources (such as wages, salaries, tips, net income from a business, interest, dividends, child support, alimony, and Social Security, public assistance, pensions, or retirement benefits).  1. Below $50,000  2. $50,000 or more  We would like to get a better estimate of your total HOUSEHOLD income in the past 12 months before taxes. Was it ... Your answer to this question is important for future surveys we will send you – your individual responses are kept confidential.  1. Less than $5,000  2. $5,000 to $7,499  3. $7,500 to $9,999  4. $10,000 to $12,499  5. $12,500 to $14,999  6. $15,000 to $19,999  7. $20,000 to $24,999  8. $25,000 to $29,999  9. $30,000 to $34,999  10. $35,000 to $39,999  11. $40,000 to $49,999  We would like to get a better estimate of your total HOUSEHOLD income in the past 12 months before taxes. Was it ... Your answer to this question is important for future surveys we will send you – your individual responses are kept confidential.  1. $50,000 to $59,999  2. $60,000 to $74,999  3. $75,000 to $84,999  4. $85,000 to $99,999  5. $100,000 to $124,999  6. $125,000 to $149,999  7. $150,000 to $174,999  8. $175,000 to $199,999  9. $200,000 to $249,999  10. $250,000 or more |
| **Race/Ethnicity** | Are you Spanish, Hispanic, or Latino?  Your answer to this question is important for future surveys we will send you – your individual responses are kept confidential.  Select all answers that apply.  1. No, I am not [S]  2. Yes, Mexican, Mexican-American, Chicano  3. Yes, Puerto Rican  4. Yes, Cuban, Cuban American  5. Yes, other Spanish, Hispanic, or Latino group (Please specify, for example Argentinean, Colombian, Dominican, Nicaraguan, Salvadoran, Spaniard, and so on)  Please indicate what you consider your race to be. We appreciate your effort to describe your background using these U.S. Census Bureau categories.  Please choose one or more race(s) that you consider yourself to be. Your answer to this question is important for future surveys we will send you – your individual responses are kept confidential.  1. White  2. Black or African American  3. American Indian or Alaska Native  4. Asian  5. Native Hawaiian or other Pacific Islander  6. Some other race |
| **Insurance coverage** | This survey is about your healthcare and healthcare coverage. Below is a list of different kinds of health insurance. Which of the following is your primary source of insurance coverage?  Select one answer only.  1. Health insurance through your or someone else’s employer or union  2. Medicare, a government plan that pays healthcare bills for people aged 65 or older and for some disabled people  3. Medicaid, or any state government medical assistance plan for those with lower incomes  4. Health insurance that you bought from the federal Health Insurance Marketplace, also known as Healthcare.gov, or a state-run Health Insurance Marketplace  5. Veteran’s Affairs (VA), Department of Defense, or other military programs  6. Health insurance from some other source  7. I do not have any healthcare insurance/coverage |
| **Medical conditions** | Have YOU been diagnosed by a doctor or other qualified medical professional with any of the following medical conditions?  high blood pressure  Asthma, chronic bronchitis, or COPD  Diabetes or pre-diabetes  Pulmonary arterial hypertension (PAH)  Nonalcoholic Fatty liver disease  Hepatitis C  HIV/AIDS  Kidney disease  Heart attack, heart disease, or other heart condition  Were you diagnosed with…?  1. Type 1 diabetes  2. Type 2 diabetes  3. Pre-diabetes or impaired glucose intolerance (IGT)  4. Not sure |
| **Have you smoked at least 100 cigarettes in your ENTIRE LIFE** | Have you smoked at least 100 cigarettes in your ENTIRE LIFE?  1. Yes  2. No |
| **To calculate BMI** | How tall are you without shoes?  Please type in the number of feet and inches separately. For example, if you are 6' 0" tall, type 6 in the feet box and 0 in the inches box.  How much do you weigh without shoes?  Please type in the number. |

**Questions from** **Equity in Health, Wealth, and Civic Engagement (English)**

Have you ever been told by a healthcare professional that you have substance use disorder or a drug addiction?

1. Yes
2. No
3. Don’t know/Not sure
4. Prefer not to answer

Have you ever been told by a healthcare professional that you have alcohol use disorder or an alcohol addiction?

1. Yes
2. No
3. Don’t know/Not sure
4. Prefer not to answer

Have you ever been told by a healthcare professional that you have opioid use disorder or an opioid addiction?

1. Yes
2. No
3. Don’t know/Not sure
4. Prefer not to answer

The following questions are about coronavirus disease or COVID-19.

Have you received a test for the Coronavirus?

1. Yes
2. No
3. Don’t know

(if working status is working as a paid employee) Do you get days of paid sick leave from your employer?

1. Yes
2. No
3. Don’t know

Have you been laid off work because of Coronavirus?

1. Yes
2. No
3. Don’t know

**Questions that are part of IPSOS Knowledge Panel (Spanish)**

| **Concept** | **Question (Spanish)** |
| --- | --- |
| **Gender** | ¿Es usted...?   \| Hombre \| \| --- \| \| Mujer \| |
| **Employment Status** | ¿Alguno de los siguientes lo describe a usted actualmente?   \| 1. Empleado a tiempo completo (35 horas o más por semana) por pago con una organización o empresa \| \| --- \| \| 1. Empleado a tiempo parcial (menos de 35 horas por semana) por pago con una organización o empresa \| \| 1. Por cuenta propia a tiempo completo (35 horas o más por semana) \| \| 1. Por cuenta propia a tiempo parcial (menos de 35 horas por semana) \|  \| 1. Sí \| \| --- \| \| 1. No \|   ¿Alguno de los siguientes lo describe a usted actualmente?   \| 1. Buscando trabajo \| \| --- \| \| 1. No puedo trabajar debido a una discapacidad o una lesión relacionada al trabajo \| \| 1. Despido temporal de un trabajo \| \| 1. Sí \| \| 1. No \|   ¿Alguno de los siguientes lo describe a usted actualmente?   \| 1. Retirado \| \| --- \| \| 1. Estudiante \| \| 1. Cónyuge o pareja que se queda en casa \| \| 1. Trabaja en un empleo no pagado, como una pasantía o una posición de voluntario \| \| 1. Trabaja por cuenta propia o como un contratista independiente por un pago \| \| 1. Sí \| \| 1. No \| |
| **Household Income** | Cuánto es el ingreso combinado de todos los INTEGRANTES DE SU HOGAR en los ÚLTIMOS 12 MESES.  Por favor incluya su ingreso MÁS el ingreso de todas las personas que viven en su hogar (incluyendo su pareja y los integrantes de las fuerzas armadas que viven en su hogar). Por favor incluya los ingresos provenientes de todas las fuentes de entrada (tales como salarios, sueldos, propinas, ingreso neto de negocios, intereses, dividendos, manutención de menores, pensión alimenticia a causa de divorcio o separación, seguro social, ayuda pública, pensiones, o beneficios de jubilación) ANTES DE PAGAR LOS IMPUESTOS.   \| 1. Menos de $50,000 \| \| --- \| \| 1. $50,000 o más \|   Nos gustaría obtener un mejor estimado del total de ingresos de su HOGAR antes de calcular los impuestos, de estos últimos 12 meses. El total de ingresos fue... Su respuesta a esta pregunta es importante para encuestas que le enviaremos en el futuro - Sus respuestas individuales se mantienen confidenciales.   \| 1. Menos de $5,000 \| \| --- \| \| 1. De $5,000 a $7,499 \| \| 1. De $7,500 a $9,999 \| \| 1. De $10,000 a $12,499 \| \| 1. De $12,500 a $14,999 \| \| 1. De $15,000 a $19,999 \| \| 1. De $20,000 a $24,999 \| \| 1. De $25,000 a $29,999 \| \| 1. De $30,000 a $34,999 \| \| 1. De $35,000 a $39,999 \| \| 1. De $40,000 a $49,999 \|   Nos gustaría obtener un mejor estimado del total de ingresos de su HOGAR antes de calcular los impuestos, de estos últimos 12 meses. El total de ingresos fue... Su respuesta a esta pregunta es importante para encuestas que le enviaremos en el futuro - Sus respuestas individuales se mantienen confidenciales.   \| 1. $50,000 a $59,999 \| \| --- \| \| 1. $60,000 a $74,999 \| \| 1. $75,000 a $84,999 \| \| 1. $85,000 a $99,999 \| \| 1. $100,000 a $124,999 \| \| 1. $125,000 a $149,999 \| \| 1. $150,000 a $174,999 \| \| 1. $175,000 a $199,999 \| \| 1. $200,000 a $249,999 \| \| 1. $250,000 o más \| |
| **Race/Ethnicity** | ¿Es usted español, hispano o latino?  Su respuesta a esta pregunta es importante para encuestas que le enviaremos en el futuro - Sus respuestas individuales se mantienen confidenciales   \| 1. No, no lo soy \| \| --- \| \| 1. Sí, mexicano, mexicano-americano, chicano \| \| 1. Sí, puertorriqueño \| \| 1. Sí, cubano, cubano-americano \| \|  \|   Por favor, indique cuál considera que es su raza. Agradecemos que intente describirla usando estas categorías de la Oficina del Censo de los Estados Unidos.  Por favor, seleccione una o más que se considere usted ser. Seleccione todas las respuestas que apliquen. Su respuesta a esta pregunta es importante para encuestas que le enviaremos en el futuro - Sus respuestas individuales se mantienen confidenciales   \| 1. Blanca \| \| --- \| \| 1. Negra o afroamericana \| \| 1. India americana o nativa de Alaska \| \| 1. Asiática \| \| 1. Nativo de Hawái, de las islas del Pacífico u otro \| \| 1. Alguna otra raza (Por favor especifique) \| |
| **Insurance coverage** | Esta encuesta trata sobre el cuidado de su salud y su cobertura de cuidado de la salud. A continuación se muestra una lista de los diferentes tipos de seguros médicos. ¿Cuál de las siguientes opciones es su principal fuente de cobertura de seguro? Seleccione sólo una respuesta   \| 1. Seguro médico a través de su empleador o el empleador de otra persona o sindicato \| \| --- \| \| 1. Medicare, un plan del gobierno que paga las cuentas del cuidado médico de personas de 65 años o mayores y para algunas personas con discapacidad \| \| 1. Medicaid o cualquier plan de ayuda médica del gobierno estatal para personas con bajos ingresos \| \| 1. Seguro médico que usted compró a través del Mercado de Seguros de Salud federal, también conocido como Healthcare.gov, o de un mercado de seguros de salud estatal \| \| 1. Asuntos de los Veteranos (VA), Departamento de Defensa, u otros programas militares \| \| 1. Un seguro médico de alguna otra fuente \| \| 1. No tengo ningún seguro médico/cobertura \| |
| **Medical conditions** | ¿Ha sido USTED diagnosticado con cualquiera de las siguientes condiciones médicas por un médico u otro profesional de la salud calificado?  Presión arterial alta  Asma, bronquitis crónica o COPD  Diabetes o pre-diabetes  Hipertensión Arterial Pulmonar (HAP)  Hígado graso no alcohólico  Hepatitis C  VIH/SIDA  Enfermedad renal  Ataque al corazón, enfermedades del corazón, u otros problemas cardíacos  ¿Usted fue diagnosticado con...?   \| 1. Diabetes tipo 1 \| \| --- \| \| 1. Diabetes tipo 2 \| \| 1. Pre-diabetes o intolerancia a la glucosa (IGT) \| \| 1. No estoy seguro(a) \| |
| **Smoking** | ¿Ha fumado por lo menos 100 cigarrillos durante TODA SU VIDA?  Si  No |
| **To calculate BMI** | ¿Cuánto mide usted sin zapatos? Por favor escriba separadamente el número de pies y pulgadas. Por ejemplo, si usted mide 6' 0", escriba el número 6 en la casilla correspondiente a los pies y 0 en la casilla correspondiente a las pulgadas.   \| Pies \| \| --- \| \| Pulgadas \|   ¿Cuánto pesa usted sin zapatos? Por favor revise su respuesta. El rango valido es de 50 a 500 libras Por favor puede confirmar que su peso indicado es el correcto.  Libras |

**Questions from Equity in Health, Wealth, and Civic Engagement (Spanish)**

¿Alguna vez le ha dicho un profesional de la salud que padece trastorno por consumo de sustancias o adicción a las drogas?

1. Sí
2. No
3. No sé / No estoy seguro
4. Prefiero no responder

¿Alguna vez le ha dicho un profesional de la salud que padece trastorno por consumo de alcohol o adicción al alcohol?

1. Sí
2. No
3. No sé / No estoy seguro
4. Prefiero no responder

¿Alguna vez le ha dicho un profesional de la salud que tiene un trastorno de consumo de opioides o una adicción a los opioides?

1. Sí
2. No
3. No sé / No estoy seguro
4. Prefiero no responder

Las siguientes preguntas son sobre la enfermedad del coronavirus o COVID-19.

¿Se ha hecho el examen del coronavirus?

Sí

No

No sé

¿Su empleador le ofrece días de licencia por enfermedad remunerada?

Sí

No

No sé

¿Le han despedido del trabajo debido al coronavirus?

Sí

No

No sé
